# Supplementary material for: Identification and functional analysis of non-coding regulatory small RNA FenSr3 in Bacillus amyloliquefaciens LPB-18
Source: PeerJ. 2023 May 15;11:e15236. doi: 10.7717/peerj.15236 (PMC10194069; doi:10.7717/peerj.15236)
Supplement: Supplemental Information 4 [file peerj-11-15236-s004.zip › KO/CK-vs-T1_map/map03410.html]

KEGG PATHWAY: Base excision repair - Reference pathway


|  |  |
| --- | --- |
| **Base excision repair - Reference pathway** |  |

[
Pathway menu
| Organism menu
| Pathway entry
| Show description
| User data mapping
]

Base excision repair (BER) is the predominant DNA damage repair pathway for the processing of small base lesions, derived from oxidation and alkylation damages. BER is normally defined as DNA repair initiated by lesion-specific DNA glycosylases and completed by either of the two sub-pathways: short-patch BER where only one nucleotide is replaced and long-patch BER where 2-13 nucleotides are replaced. Each sub-pathway of BER relies on the formation of protein complexes that assemble at the site of the DNA lesion and facilitate repair in a coordinated fashion. This process of complex formation appears to provide an increase in specificity and efficiency to the BER pathway, thereby facilitating the maintenance of genome integrity by preventing the accumulation of highly toxic repair intermediates.

|  |  |  |
| --- | --- | --- |
| Reference pathway | 184% 150% 122% 100% 82% 67% 55% | 图片下载 |
